# Vitamin D Levels in Different Severity Groups of Schizophrenia

**DOI:** 10.3389/fpsyt.2017.00105

**Published:** 2017-06-13

**Authors:** Kehinde Sola Akinlade, Oyejide Afolabi Olaniyan, Victor Olufolahan Lasebikan, Sheu Kadiri Rahamon

**Affiliations:** ^1^Metabolic Research Unit, Department of Chemical Pathology, University College Hospital and University of Ibadan, Ibadan, Nigeria; ^2^Department of Chemical Pathology, University of Ibadan, Ibadan, Nigeria; ^3^Department of Psychiatry, University College Hospital and University of Ibadan, Ibadan, Nigeria

**Keywords:** antipsychotics, vitamin D, Positive and Negative Syndrome Scale, poor nutrition, schizophrenia severity

## Abstract

**Background:**

Vitamin D deficiency (VDD) continues to be associated with schizophrenia, but there is the dearth of information on the relationship between the severity of schizophrenia and plasma levels of vitamin D. This study, therefore, determined the plasma levels of vitamin D in different severity groups of schizophrenia.

**Materials and methods:**

Plasma level of vitamin D was determined in 60 patients with schizophrenia and 30 apparently healthy individuals who served as controls. Patients with schizophrenia were classified into mildly ill, moderately ill, markedly ill, and severely ill groups using the Positive and Negative Syndrome Scale (PANSS).

**Results:**

The mean level of vitamin D was significantly lower in patients with schizophrenia compared with the controls. Similarly, there was a significant association between VDD and schizophrenia. The mean plasma levels of vitamin D were not significantly different when the mildly, moderately, markedly, and severely ill groups were compared with one another and there was no significant correlation between vitamin D level and PANSS scores. Furthermore, patients on atypical antipsychotics had an insignificantly lower level of vitamin D compared with the patients on typical antipsychotics.

**Conclusion:**

It could be concluded from this study that patients with schizophrenia have low plasma vitamin D level which does not appear to be associated with the severity of schizophrenia and type of antipsychotics. Therefore, regular screening for vitamin D status of patients with schizophrenia is suggested in order to allow for the institution of appropriate clinical intervention when necessary.

## Introduction

Vitamin D is a fat soluble pleotropic steroid hormone which is synthesized in the skin on exposure to ultraviolet light or obtained from the diet (1). Although its role in maintenance of calcium homeostasis and bone mineral density are well known (1, 2), its link with myriads of clinical conditions such as cardiovascular diseases, infection, vaso-occlusive crisis, diabetes, and mental disorders continues to generate research interests of public health importance (3–6).

Emerging evidences show that vitamin D is a neuroactive hormone that plays important roles in brain development and function (7, 8). The Third National Health and Nutrition Examination Survey (NHANES III) as well as other reports showed that vitamin D deficiency (VDD) is associated with increased odd of poor cognition (9–11). A similar observation was reported by Annweiler et al. (12) in women with inadequate vitamin D intake.

Generally, patients with psychosis are more likely to have VDD but it is now elucidated that schizophrenics have a trend for lower levels than other forms of psychosis (10, 13). This trend in schizophrenics was brought to the fore in two large infant cohort studies which showed that low vitamin D level significantly increases the risk of schizophrenia with the risk being significantly reduced in infants with adequate vitamin D level and those on vitamin D supplementation (14–16). Graham et al. (8) also showed that low vitamin D level is associated with severe cognitive deficits and low verbal fluency in adults with schizophrenia. This deficiency is shown not to be associated with antipsychotic medications (17).

Presently, the possible reason for VDD in schizophrenics is poorly understood but certain social and lifestyle factors are implicated. For example, social isolation and amotivation can lead to poorer nutrition while low sun exposure due to less time spent outdoors can, independently or mutually, cause VDD in patients with schizophrenia (8). Although reports on vitamin D level in patients with schizophrenia are available, there is the dearth of information on the relationship between the severity of schizophrenia and plasma levels of vitamin D. Therefore, this study determined the plasma levels of vitamin D in different severity groups of schizophrenia.

## Materials and Methods

### Study Center

The study was carried out at the New World Psychiatric Hospital, Ibadan, in June 2015. The study center is a private psychiatric hospital that offers both inpatient and outpatient services and located in the South Western part of Nigeria.

### Study Participants

A total of 90 participants (aged between 19 and 55 years) comprising 60 patients with schizophrenia and 30 apparently healthy individuals (non-biological caregivers) with no history of psychosis, who served as controls, were enrolled in this preliminary study. The controls were matched by age with ratio two patients to one control.

### Diagnosis of Schizophrenia

For patients to be eligible for the study, they were expected to meet the ICD 10 criteria for schizophrenia. This was achieved by using the Mini International Neuropsychiatric Interview (MINI Plus) Version 5.0. The MINI Plus (5.0) was developed from the MINI (18) as an efficient diagnostic interview, to be used in clinical and research settings, and follows *DSM-IV* and ICD-10 criteria. The MINI has cross-cultural reliability and validity and has previously been used in Nigeria (19).

An additional eligibility criterion was that patients were expected to be on a monotherapy of either an atypical or a typical antipsychotic medication.

### Exclusion Criteria

We excluded patients who had severe and unstable general medical conditions and those with any other ICD 10 psychiatric diagnoses or psychoactive substance use disorder. We also excluded those who were younger than 18 years.

### Ethical Consideration

All the study participants were enrolled after an approval from the University of Ibadan/University College Hospital (UI/UCH) Joint Ethics Committee. Also, written informed consent was obtained from each participant or otherwise assents from their appropriate relative or guardian.

### Measurement of Anthropometric Indices and Blood Pressure

Height, body weight, waist circumference, and blood pressure were measured using standard methods. Thereafter, body mass index (BMI) and waist-to-hip ratio (WHR) were calculated appropriately.

### Sample Collection and Storage

Five milliliters of venous blood were collected from each participant into lithium heparin bottles. Plasma was appropriately obtained and stored at −20°C until analyzed.

### Estimation of Vitamin D

Plasma levels of vitamin D were determined using ELISA (WKEA Med Supplies, China).

### Classification of Vitamin D

Plasma vitamin D level was classified as reported by Holick (3). Levels of vitamin D ≤20, 21–29, ≥30–150, and >150 ng/ml were considered as VDD, vitamin D insufficiency, vitamin D sufficiency, and vitamin D intoxication, respectively.

### Severity Classification of Schizophrenia

Patients with schizophrenia were classified into different severity groups using the Positive and Negative Syndrome Scale (PANSS). The PANSS is a 30-item scale, 7-point rating instrument, adapted from the Brief Psychiatric Rating Scale (20) and 12 items from the Psychopathology Rating Schedule (21). Each item on the PANSS is scored from 1 to 7, indicating increasing levels of psychopathology, with 1 denoting absence to 7 denoting extreme symptoms. Of the 30 psychiatric parameters assessed on the PANSS, 7 constitutes a Positive Scale, 7 make up a Negative Scale, and the remaining 16 General Psychopathology. PANSS scores of 58, 75, 95, and 116 were classified as mildly ill, moderately ill, markedly ill, and severely ill, respectively.

### Statistical Analysis

Results are expressed as mean ± SD or number (percentages). ANOVA and the independent Student’s *t*-test were used to compare differences in means of the variables. Association between dichotomous variables was determined using Chi-square test. Also, Pearson’s correlation was used to determine the relationship between the variables. *P*-values less than 0.05 were considered as statistically significant.

## Results

Table 1 shows the characteristics of the study participants. The mean pulse was significantly higher while the mean levels of SBP and vitamin D were significantly lower in patients with schizophrenia when compared with the controls.

**Table 1:** Characteristics and levels of vitamin D in the study participants.

Parameters	Schizophrenia (*n* = 60)	Control (*n* = 30)	*P*-value
Age (years)	35.10 ± 9.15	35.27 ± 7.97	0.933
Height (m)	1.71 ± 0.99	1.68 ± 0.08	0.130
Body weight (kg)	70.50 ± 16.51	67.90 ± 12.59	0.450
BMI (kg/m^2^)	24.00 ± 4.59	24.36 ± 5.37	0.738
WC (cm)	83.67 ± 12.55	87.57 ± 13.37	0.178
HC (cm)	93.00 ± 17.77	91.73 ± 12.68	0.728
WHR	0.93 ± 0.30	0.97 ± 0.16	0.521
SBP (mmHg)	113.03 ± 14.26	124.67 ± 16.13	0.001*
DBP (mmHg)	74.75 ± 10.09	77.63 ± 9.67	0.198
Pulse (s)	87.75 ± 16.70	76.73 ± 10.77	0.003*
Vitamin D (ng/ml)	19.75 ± 5.19	28.06 ± 5.63	0.000*

*Significant at P < 0.05.

*BMI, body mass index; WC, waist circumference; HC, hip circumference; WHR, waist hip ratio; SBP, systolic blood pressure; DBP, diastolic blood pressure*.

The vitamin D status of the study participants is presented in Table 2. The proportion of patients with VDD was significantly higher in patients with schizophrenia compared with the controls. Only 1 (1.7%) patient with schizophrenia had vitamin D sufficiency compared with 11 (36.7%) controls who had sufficient vitamin D level. However, the majority (56.7%) of the controls had vitamin D insufficiency but none of the study participants had vitamin D intoxication.

**Table 2:** Vitamin D status of the study participants.

	Schizophrenia	Control	*n*	χ^2^	*P*-value
Vitamin D sufficiency	1 (1.7%)	11 (36.7%)	12	32.581	0.000*
Vitamin D insufficiency	24 (40.0%)	17 (56.7%)	41		
Vitamin D deficiency	35 (58.3%)	2 (6.7%)	37		

*Significant at P < 0.05.

**Figure 1:**
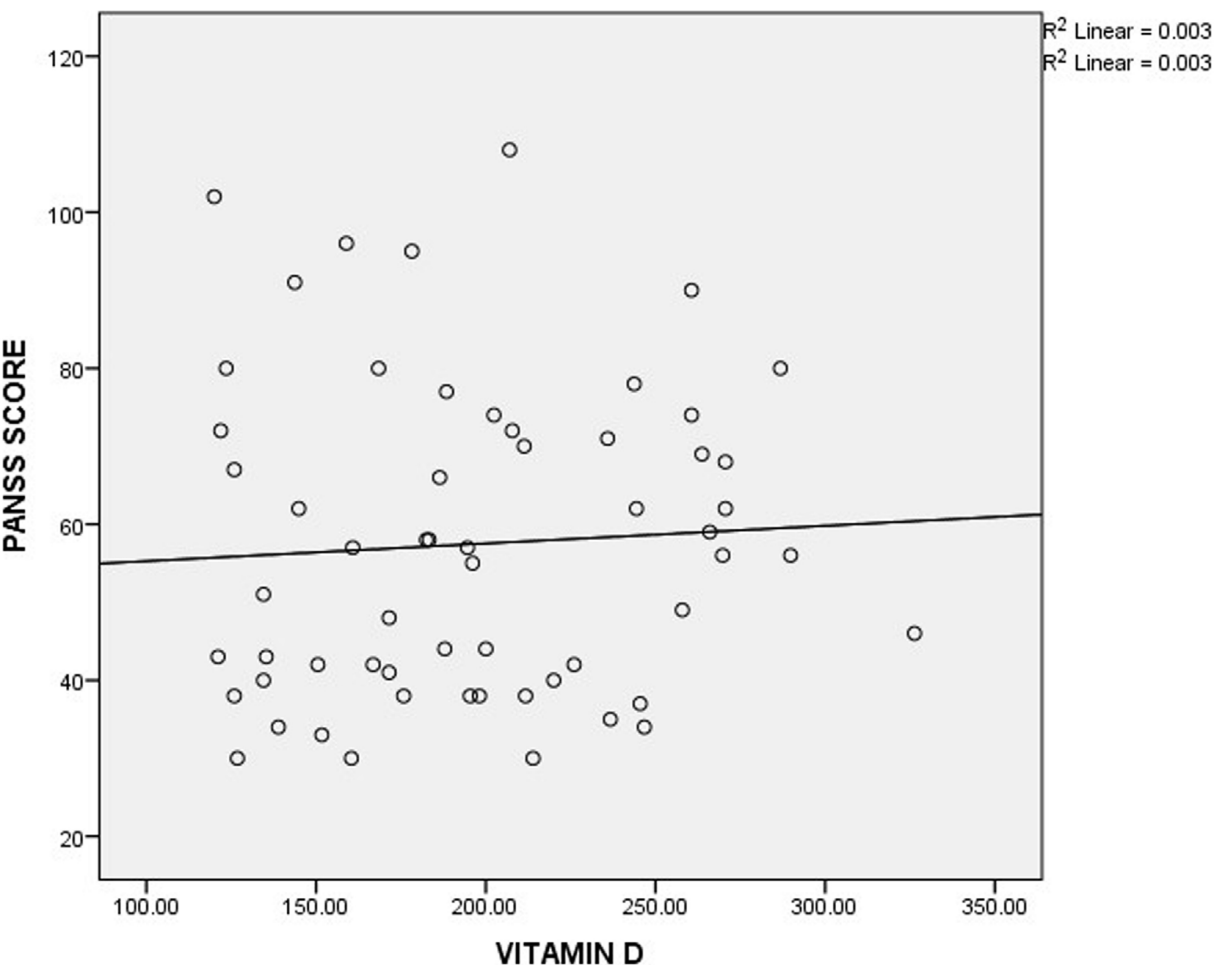
Correlation between plasma level of vitamin D and Positive and Negative Syndrome Scale (PANSS) scores.

As shown in Table 3, the mean plasma levels of vitamin D were insignificantly different in mildly, moderately, markedly, and severely ill patients, when compared with one another using ANOVA (*P*-value = 0.329). To further understand the relationship between schizophrenia severity and plasma level of vitamin D, the patients were divided into two groups based on the mean score (57.46) obtained from the PANSS. The vitamin D level in patients with PANSS scores greater than or equal to the mean is similar to those with PANSS scores less than the mean (Table 4). Similarly, there was no significant correlation between vitamin D level and PANSS scores (*r*-value = 0.059, *P*-value = 0.658) (Figure 1).

**Table 3:** Plasma levels of vitamin D in different severity classes of schizophrenia.

Severity classes of schizophrenia	Number of participants	Vitamin D levels (ng/ml)	*P*-value
Mildly ill	34	19.14 ± 4.99	0.149
Moderately ill	14	21.52 ± 5.34	
Mildly ill	34	19.14 ± 4.99	0.893
Markedly and Severely ill	12	18.90 ± 5.53	
Moderately ill	14	21.52 ± 5.34	0.247
Markedly and Severely ill	12	18.90 ± 5.53	

**Table 4:** Vitamin D status based on PANSS score and the type of antipsychotics.

	Number of patients	Mean ± SD (ng/ml)	*P*-value
**PANSS scores**			
<Mean	32	19.20 ± 5.15	0.382
≥Mean	28	20.38 ± 5.26	
**Type of antipsychotic**			
Typical	34	20.20 ± 5.30	0.629
Atypical	26	19.51 ± 4.91	

*PANSS, Positive and Negative Syndrome Scale*.

*Mean score of PANSS = 57.46*.

The possible effect of antipsychotics on vitamin D level was also assessed. It was observed that patients on atypical antipsychotics had an insignificantly lower level of vitamin D compared with those on typical antipsychotics (Table 4).

## Discussion

Vitamin D sufficiency is critical for the maintenance of mental function with major impact on the hippocampus, thalamus, hypothalamus, amygdala, the prefrontal cortex, and the temporal lobe (22, 23). The deficiency of vitamin D continues to be associated with an increased incidence of schizophrenia (24, 25).

The observed significantly lower level of vitamin D in patients with schizophrenia as well as its significant association with schizophrenia compared with the controls support earlier reports (26, 27). This observation could be due to poor nutrition and inadequate sun exposure in patients with psychosis (8, 28). Also, it is possible that some of the drugs used in the treatment of schizophrenia interfere with the normal metabolism of vitamin D. Tangpricha and Khazai (29) reported that drugs such as Dilantin, phenobarbital, and rifampin induce hepatic p450 enzymes that accelerate vitamin D catabolism. Furthermore, an *in vitro* study showed that antipsychotics impair vitamin D synthesis (30). Irrespective of the etiology of the lower level of vitamin D observed in this study, there is an indication that patients with schizophrenia are at risk of disorders associated with VDD. Apart from the well-reported adverse metabolic and cardiovascular effects of VDD, it has been shown to impair neuronal growth and development via reduction in protein kinase B activity. It also causes cellular hyperpolarization, memory and attention deficits, hyperlocomotion, and problems in cognitive function (23, 31–33). Therefore, there is a need for regular assessment of vitamin D level in patients with schizophrenia with a view to identifying those who would benefit from appropriate clinical intervention including vitamin D supplementation. Since the body pool of vitamin D can be affected by myriads of factors, studies that will determine whether VDD in patients with schizophrenia is due to, either or a combination of, psychosocial factors associated with schizophrenia, antipsychosis, or schizophrenia itself are suggested.

Definition of vitamin D insufficiency and deficiency continues to be a matter of debate as criterion for optimal vitamin D status has moved away from that required to achieve skeletal health to that with optimal benefits on non-skeletal health outcomes (34). However, a cut-off value of 30 ng/ml is usually considered as optimal vitamin D status due to the observation that at 30 ng/ml of 25-hydroxyl vitamin D, a plateau in suppression of parathyroid hormone is demonstrated (35). The observed high proportion of controls with vitamin D insufficiency is of public importance as it indicates that majority of apparently healthy Nigerians are likely to be insufficient or even deficient of vitamin D. Although our observation could be due to the classification of Holick (whose bar for vitamin D status is considered high) used in this study, there is still an urgent need for a population study that will reveal the true vitamin D status of Nigerians and come up with necessary suggestions such as supplementation with a view to prevent disorders associated with VDD.

Evaluation of symptom severity as well as positive and negative symptoms in patients with schizophrenia is usually done using scores obtained from PANSS. The observed insignificant differences in vitamin D levels across the severity classification groups might suggest that there is no direct association between plasma level of vitamin D and schizophrenia severity. This observation is further buttressed by the observed insignificant correlation between the plasma level of vitamin D and the scores obtained from PANSS. A similar observation was reported by Itzhaky et al. (26) where they showed that vitamin D level had no correlation with schizophrenia severity.

It is well known that both typical and atypical antipsychotics cause metabolic alteration (36, 37). An experimental study showed that this metabolic alteration can be aggravated by VDD while it can be alleviated by vitamin D supplementation (38). The observed insignificantly lower level of vitamin D in patients on atypical antipsychotics compared with patients on typical antipsychotics indicates that there seems to be no differential effect of the type of antipsychotics on the plasma level of vitamin D. Our observation, however, needs a confirmation through a large population study as the small sample size used for this study was a major limitation.

Hypotension and hypertension are both associated with schizophrenia and they are responsible for a higher cardiac mortality rate in patients with schizophrenia (39). The observed significantly higher pulse rate and lower blood pressure in patients with schizophrenia compared with controls suggests tachycardia and hypotension which might be reflections of some of the side effects of antipsychotics the patients are taking. Antipsychotics especially, low potency typicals and clozapine, are known to cause hypotension via antagonism of alpha-1-adrenoreceptor. Therefore, patients with schizophrenia on treatment should be monitored closely to prevent risks such as syncope, falls, and orthostatic hypotension among others (39, 40).

It could be concluded from this study that patients with schizophrenia have low plasma vitamin D level, which does not appear to be associated with the severity of schizophrenia and type of antipsychotics. Therefore, regular screening for vitamin D status of patients with schizophrenia is suggested in order to allow for the institution of appropriate clinical intervention when necessary.

## Ethics Statement

All the study participants were enrolled after an approval from the University of Ibadan/University College Hospital (UI/UCH) Joint Ethics Committee. Also, written informed consent was obtained from each participant or otherwise assents from their appropriate relative or guardian.

## Author Contributions

KA designed the study, VL made the diagnosis, and all authors recruited the patients and wrote the manuscript. KA and SR supervised the entire research.

## Conflict of Interest Statement

The authors declare that the research was conducted in the absence of any commercial or financial relationships that could be construed as a potential conflict of interest.
